# Japan’s Position in the Global Standard to Ban Tobacco Advertising in the Media

**DOI:** 10.2188/jea.JE20220074

**Published:** 2022-07-05

**Authors:** Masao Ichikawa, Takahiro Tabuchi

**Affiliations:** 1 Department of Global Public Health, Faculty of Medicine, University of Tsukuba, Ibaraki, Japan; 2 Cancer Control Center, Osaka International Cancer Institute, Osaka, Japan

**Keywords:** advertising, comparison, legislation, tobacco

A ban on tobacco advertising is crucial for tobacco control because exposure to such advertisements increases the risk of smoking initiation and habituation.^1^ Enforcement of the ban is one of the six tobacco control measures proposed by the World Health Organization (WHO) in the provisions of the WHO Framework Convention on Tobacco Control, which has been widely adopted irrespective of the country’s economic level.^2^ In February 2022, Switzerland—the headquarters of major tobacco companies, including Japan Tobacco International—finally decided to ban tobacco advertising from 2023, following a referendum where 57% of voters supported it.^3^ Meanwhile, public debate on this issue is lacking in Japan, where tobacco advertising is only self-regulated by tobacco companies at their discretion. To spark the debate, we compared Japan with other countries worldwide in terms of banning tobacco advertising in the media.

We obtained information on the countries’ ban on tobacco advertising in the media from the WHO’s Global Health Observatory.^4^ As of February 2022, the information was available for the status of “ban” in 2018, which was last updated in May 2020. The information was available for 195 countries, including 192 of 193 United Nations member states (excluding Liechtenstein) and three nonmember states (the Cook Islands, Niue, and Palestine). We calculated the proportion of countries banning tobacco advertising in four types of media (domestic newspapers and magazines, domestic television and radio, the Internet, and billboards and outdoor advertising) by six WHO regions (Africa, Americas, Eastern Mediterranean, Europe, South-East Asia, and Western Pacific). Domestic television and radio include cable television if the signal originates in the country. Advertising on television and radio was not considered banned if it was restricted to some hours during the day or a type of audience, such as children. Advertising on the Internet included contextual ads, banner ads, advertising networks, and e-mail marketing. Billboards and outdoor advertising included any sign, posting, or other outdoor advertising. Furthermore, to show the geographical distribution of the countries banning tobacco advertising, we created map charts using Microsoft Excel (Microsoft Corp., Redmond, WA, USA).

**Figure 1.**
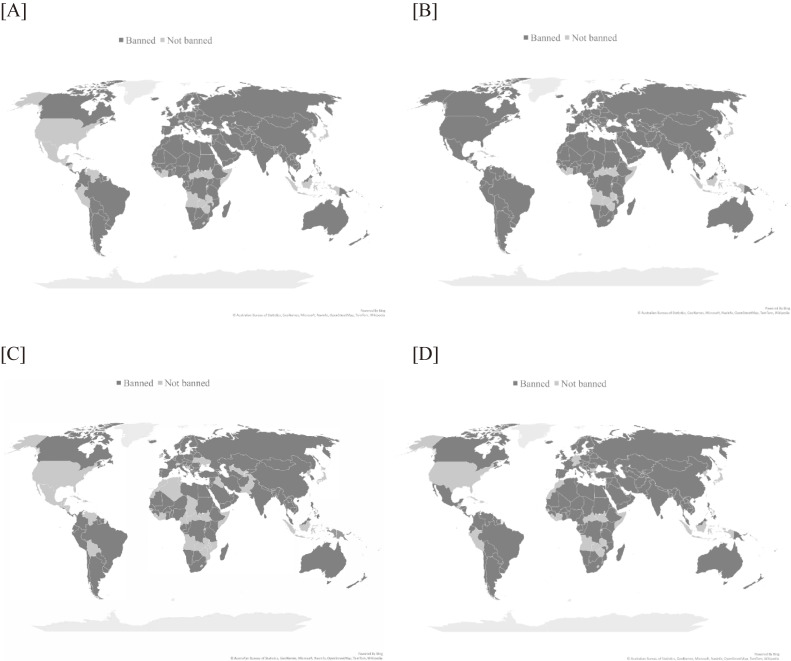
Countries banning tobacco advertising in four types of media as of 2018: [A] domestic newspapers and magazines, [B] domestic television and radio, [C] the Internet, and [D] billboards and outdoor advertising. Map charts were created in Microsoft Excel powered by Bing (© Australian Bureau of Statistics, GeoNames, Microsoft, Navinfo, OpenStreetMap, TomTom, Wikipedia).

Table 1 shows the number and proportion of countries banning tobacco advertising in four types of media by region. Of the 195 counties, 77%, 84%, 68%, and 79% banned tobacco advertising in domestic newspapers and magazines, domestic television and radio, the Internet, and billboards and outdoor advertising, respectively. These proportions were lowest in the American region among all six regions. While 126 countries (65%) worldwide banned tobacco advertising in all four types of media, 30 countries (15%) did not ban it in any of these media. Notably, 21 of the 30 countries were parties to the WHO Framework Convention on Tobacco Control, with Japan being one of them; only one of these countries is in the Western Pacific region. Figure 1 shows the geographical distribution of countries banning tobacco advertising in four types of media, where Japan appears to be prominent in its region.

**Table 1.** Number and proportion of countries banning tobacco advertising in four types of media by region, as of 2018

Region (number of countries)	Domestic newspapers and magazines		Domestic television and radio		Internet		Billboards and outdoor advertising		All media		Countries not banning tobacco advertising in all media (number of countries)
Africa (47)	33	70%	34	72%	28	60%	34	72%	28	60%	Angola, Central African Republic, Côte d’Ivoire, Equatorial Guinea, Guinea-Bissau, Lesotho, Liberia, Malawi^a^, Rwanda, Sierra Leone, South Sudan^a^, Zambia, Zimbabwe (13)
Americas (35)	16	46%	24	69%	15	43%	20	57%	14	40%	Barbados, Belize, Cuba^a^, Dominica, Dominican Republic^a^, Grenada, Guatemala, Haiti^a^, Saint Kitts and Nevis, Saint Lucia, Saint Vincent and the Grenadines (11)
Eastern Mediterranean (22)	21	95%	21	95%	17	77%	20	91%	17	77%	Somalia^a^ (1)
Europe (53)	48	91%	51	96%	43	81%	46	87%	39	74%	Andorra^a^, Monaco^a^ (2)
South-East Asia (11)	9	82%	9	82%	8	73%	9	82%	8	73%	Democratic People’s Republic of Korea, Indonesia^a^ (2)
Western Pacific (27)	24	89%	25	93%	21	78%	26	96%	20	74%	Japan (1)

Total (195)	151	77%	164	84%	132	68%	155	79%	126	65%	

^a^Non-parties to the World Health Organization (WHO) Framework Convention on Tobacco Control.

Among the six tobacco control measures (monitoring tobacco use; protecting people from tobacco smoke; quitting tobacco; warning about the dangers of tobacco; enforcing bans on tobacco advertising, promotion, and sponsorship; and raising taxes on tobacco) proposed in 2008, Japan has made no achievements in enforcing the advertisement ban.^5^ Consequently, tobacco advertisements still persistently appear in mass media, rendering the marketing strategies of tobacco companies in self-regulating their ads insufficient. People have no choice but to come across the ads, even if they wish to avoid them for children. However, it is not too late for Japan to follow the footsteps of Switzerland and the other 164 countries that ban tobacco advertisements.
